# *N*-Carbazolyl π-Radical and Its Antiaromatic Nitrenium Ion: A Threshold Photoelectron Spectroscopic Study

**DOI:** 10.1021/acs.jpca.4c05855

**Published:** 2024-10-26

**Authors:** Mayank Saraswat, Adrian Portela-Gonzalez, Enrique Mendez-Vega, Wolfram Sander, Patrick Hemberger

**Affiliations:** † Lehrstuhl für Organische Chemie II, Ruhr-Universität Bochum, Bochum 44780, Germany; ‡ Laboratory for Synchrotron Radiation and Femtochemistry, Paul Scherrer Institut (PSI), Villigen CH-5232, Switzerland

## Abstract

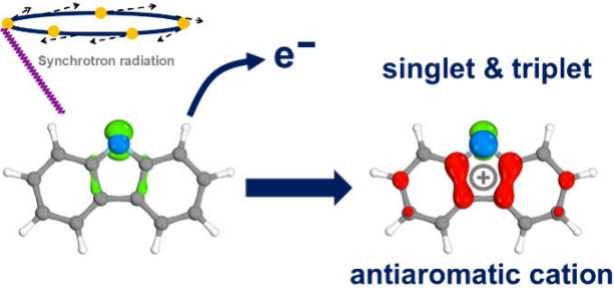

Understanding the structure and properties of heterocyclic radicals and their cations is crucial for elucidating reaction mechanisms as they serve as versatile synthetic intermediates. In this work, the *N*-carbazolyl radical **1** was generated via pyrolysis and characterized using photoion mass-selected threshold photoelectron spectroscopy coupled with tunable vacuum-ultraviolet synchrotron radiation. The *N*-centered radical **1** is classified as a π-radical (^2^B_1_), with the unpaired electron found to be delocalized over the central five-membered ring of the carbazole. Adiabatic ionization energies corresponding to the transition from radical **1** to its singlet **1**^+^(^1^A_1_) and triplet **1**^+^(^3^B_2_) cations were determined to be 7.70 ± 0.03 and 8.14 ± 0.03 eV, respectively. The antiaromatic nitrenium ion **1**^**+**^ exhibits a singlet ground state with an experimental singlet–triplet energy gap (Δ*E*_S–T_) of −0.44 eV (10.1 kcal/mol), in very good agreement with theory. *N*-centered radicals are found to have a higher ionization energy than their *C*-centered analogues due to stabilization of the singly occupied molecular orbital.

## Introduction

Nitrenium ions (R_2_N)^+^ are divalent, highly reactive, and electron-deficient nitrogen species that are isoelectronic with carbenium cations (R_2_CH^+^).^1^ Nitrenium ions are fascinating intermediates not only due to their utility in organic synthesis but also because of their role in DNA damage.^2^ It has been proposed that the enzymatic activation of aromatic arylamines involves arylnitrenium ions which, under certain circumstances, can turn healthy cells into cancer cells.^3^ Thus, nitrenium ions are considered critical intermediates in some biological systems due to their chemical toxicology.^4^ Nitrenium ions can be generated via ionization of respective *N*-centered radicals but also by protonation of monovalent nitrenes.^5^

Nitrogen-centered free radicals (NCRs) are the neutral analogues to nitrenium ions and are fundamentally important reactive intermediates in various chemical and biological settings.^6^ The generation and characterization of these intermediates are experimentally challenging due to their reactivity induced by their large electrophilicity.^7,8^ Among the free radicals, heterocyclic radicals gain significant importance due to their roles in biological,^9−11^ medicinal,^12,13^ agrochemical, material,^14^ polymer,^15,16^ atmospheric chemistry,^17,18^ and astrochemistry.^19−21^

**Scheme 1:**
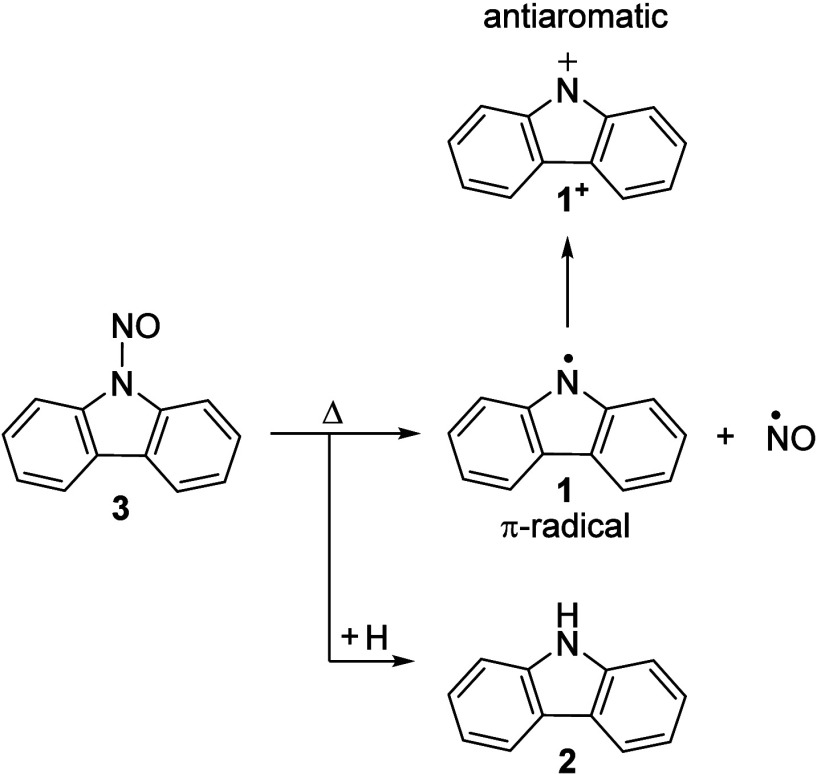
Pyrolytic Generation of the *N*-Carbazolyl Radical **1**

Nitrogen-centered radicals (NCRs) have a long history similar to classical and more popular carbon-based radicals in synthetic chemistry, but are by far much less well understood.^22^ Depending on various factors such as conjugation, delocalization, substitutions, and *N*-hybridization, NCRs can serve as nucleophiles or electrophiles and hold much potential as useful amphiphilic synthetic intermediates.^23−26^ In the past decades, visible-light photoredox catalysis has emerged as a powerful tool in synthetic organic chemistry to generate a diverse range of NCRs in a controlled fashion under mild conditions via a single electron transfer process.^27−30^ The *N*-carbazolyl radical **1** (Scheme 1) has been utilized in molecules with thermally activated delayed fluorescence (TADF) properties,^31^ optoelectronic materials,^32^ and as chromophores for fluorescent probes in imaging applications.^33^ Carbazole is the simplest core of nitrogen-substituted polycyclic aromatic hydrocarbons (PANHs), which are postulated for their contribution in diffuse interstellar bands.^34−36^ Previously carbazolyl radical **1** was studied experimentally in low-temperature argon matrices^37^ whereas its anion, carbazolide **1**^**–**^, was examined using cryogenic photodetachment spectroscopy.^38^ Furthermore, the carbazolyl nitrenium ion was studied in solution by Falvey et al. using laser flash photolysis, chemical trapping experiments, and product analysis and addressed the degree of antiaromaticity for the nitrenium ion. This makes the carbazolyl radical **1** and its nitrenium cation (**1**^**+**^) compelling subjects to explore their electronic structure utilizing photoionization.^39^ Thus, the goal of this study is to measure the experimental threshold photoelectron spectrum, determine the adiabatic ionization energy and the singlet–triplet gap, and relate these thermochemical properties to computations and structurally similar hydrocarbon radicals.

Photoionization with tunable VUV synchrotron radiation has been widely utilized in the past decades to disentangle complex chemical mixtures in heterogeneous catalysis,^40,41^ combustion,^42^ or astrochemical settings,^35^ thanks to the difference in the ionization energies of isomers.^43,44^ Double imaging photoelectron photoion coincidence spectroscopy (i^2^PEPICO) is a versatile method to identify and unveil the electronic, geometrical, and vibrational structures of neutral and charged reactive intermediates due to its mass- and isomer-selectivity.^45,46^ Since the numbers of isomers are dramatically increasing with the number of atoms in a molecule, it is challenging to assign the right species. Understanding the influence of the heteroatom substitution on the electronic structure and the adiabatic ionization energy can help to shrink the computational burden to predict the right isomer.^47^

Herein, we *in situ* generate the carbazolyl radical **1** via pyrolysis in a Chen-type reactor^48^ and study its photoionization using vacuum-ultraviolet (VUV) synchrotron radiation from the Swiss Light Source. Mass-selected threshold photoelectron (ms-TPE) spectroscopy was employed as an analytical tool to detect and spectroscopically characterize radical **1**, as well as to experimentally obtain its ionization energy, and unveil the electronic structure of its corresponding antiaromatic nitrenium ion (Scheme 1).

## Methods

The experiments were performed at the VUV beamline of the Swiss Light Source (SLS) located at Paul Scherrer Institute, Switzerland.^49^ The double imaging photoelectron photoion coincidence (i^2^PEPICO) endstation was utilized for data collection.^50^*N*-Nitrosocarbazole (**3**) was heated to 50 °C and expanded through a nozzle with a constant flow of He into a pyrolysis reactor (SiC tube, 1 mm inner diameter and 15 mm heated length). The pyrolysis reactor was electrically heated in the range of 500–800 K, and the resulting molecular beam entered the experimental chamber through a skimmer (2 mm). Upon ionization using VUV synchrotron radiation in the experimental chamber, photoions and photoelectrons were accelerated in opposite directions followed by coincident detection using position sensitive delay line anode Roentdeck DL40 detectors. The time-of-flight (TOF) mass spectrum was recorded by determining the time difference between the coincident photoions and the photoelectrons. The ms-TPE spectral data were recorded by scanning the photon energy from 7.0 to 9.0 eV and plotted against the targeted and fixed *m*/*z* value. The false coincidence as well as the hot (kinetic energy) electron background was subtracted during the analysis.^51^

Geometries and vibrational frequencies of the neutral and cationic species in their ground and excited states were calculated at the M06-2X/6-311++G** level of theory using Gaussian 16^52^ and Turbomole.^53^ Additionally, adiabatic ionization energies (AIEs), the singlet–triplet energy gap (Δ*E*_ST_), and higher energy excited states were also computed using CBS-QB3 and G4 composite methods,^54^ CCSD(T)/aug-cc-pV(D/T)Z, and multiconfigurational CASSCF and NEVPT2 methods. The complete active space CAS(e^–^, MOs) includes 14 orbitals (relevant σ, π, p, and π*) and 15 e^–^ for **1** and 14 e^–^ for **1**^**+**^, denoted CASSCF(15/14,14). Coupled cluster and multiconfigurational calculations were conducted with Molpro 2012.^55^ Franck–Condon (FC) simulations at 0 and 800 K were performed from optimized geometries and vibrational normal modes obtained with M06-2X/6-311++G** using Gaussian 16.^52^ The stick spectra were subsequently convoluted with a Gaussian function (full width at half-maximum, FWHM = 25 meV).

## Results and Discussion

### Mass Spectra of FVP Products

**Figure 1:**
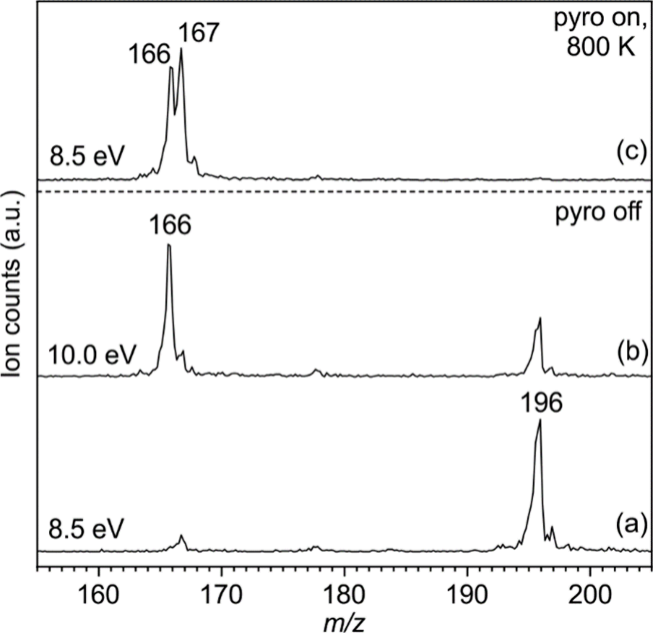
Time-of-flight mass spectra of *N*-nitrosocarbazole (**3**) recorded at RT (pyrolysis off) with (a) 8.5 and 10.0 eV and upon FVP at 800 K with (c) 8.5 eV.

*N*-Nitrosocarbazole (**3**), with a thermally labile N–NO bond with an estimated bond dissociation energy of 31.9 kcal/mol at M06-2X/6-311++G**, was employed as a thermal precursor for *N*-carbazolyl radical **1**. Pyrolysis conditions were optimized using photoionization mass spectrometry (MS) to increase the selectivity toward radical **1** (*m*/*z* 166) by recording mass spectra at different photon energies (Figure 1). Mass spectra recorded at room temperature (pyrolysis off) at a photon energy of 8.5 eV show an intense peak at *m*/*z* 196, corresponding to ionized precursor **3**^**+**^ (Figure 1a). Upon increasing the photon energy to 10.0 eV, a new peak at *m*/*z* 166 appears due to dissociative photoionization (DPI) of precursor **3** via NO loss (Figure 1b). The signal at *m*/*z* 166 is assigned to nitrenium ion **1**^**+**^. At a fixed photon energy of 8.5 eV and 800 K, precursor **3** is completely consumed leading to the maximum yield of radical **1** (*m*/*z* 166). The thermal generation of radical **1** is confirmed by the narrow velocity distribution of the molecules at *m*/*z* = 166 under these conditions (Figure S1). Additionally, a peak at *m*/*z* 167 corresponding to ionized carbazole **2**^**+**^ is also observed (Figure 1c). Radical **1** is highly reactive and can abstract a hydrogen atom and form carbazole **2** in the pyrolysis tube via bimolecular reactions in the gas phase.^56^ Radical **1** and carbazole **2** are unequivocally identified by using mass-selected threshold photoelectron spectroscopy, as discussed in the next section.

### ms-TPE Spectra of FVP Products

**Figure 2:**
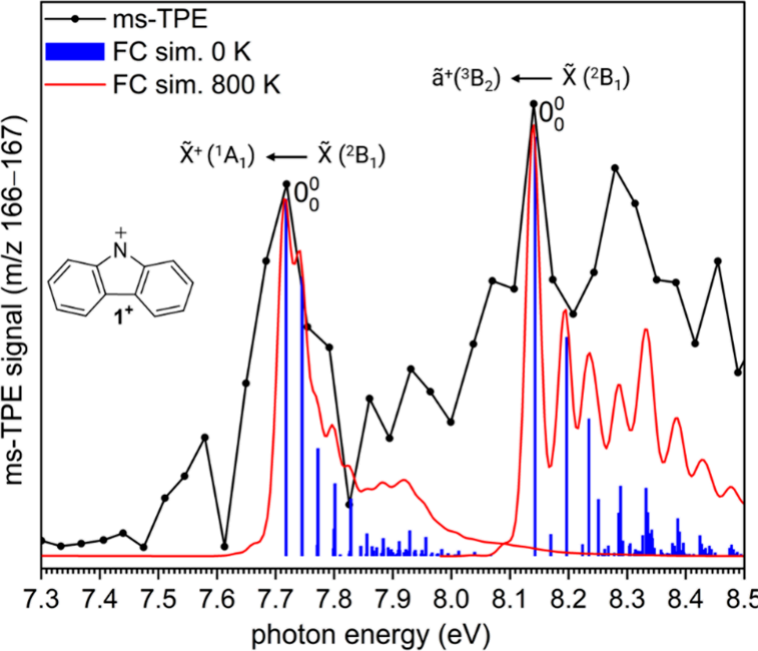
Comparison of the ms-TPE spectra of the signals at *m*/*z* 166 recorded upon FVP of precursor **3** at 800 K, with Franck–Condon (FC) simulations of carbazolyl radical **1** to cation **1**^**+**^ in its ^1^A_1_ and ^3^B_2_ electronic states. FC simulations were performed at 0 K (blue sticks) and 800 K (red trace) by convolution with 25 meV FWHM Gaussians over the vibrational frequencies computed with M06-2X/6-311++G**.

The ms-TPE spectra of molecules with *m*/*z* = 166 (C_12_H_8_N) and 167 (C_12_H_9_N), generated from pyrolysis of precursor at 800 K, are shown in Figure 2 and Figure S2, respectively. The signal at *m*/*z* 166 partially overlaps with the slightly larger signal at *m*/*z* 167, which was present as contamination of the precursor or formed upon efficient H-abstraction by the *N*-carbazolyl radical **1** after collision with the chamber or reactor walls, yielding carbazole **2** (Figure 1c). The ms-TPE signal at *m*/*z* 166 is partially contaminated by the ms-TPE signal at *m*/*z* 167, which is taken care of by using a subtraction scheme as shown (Figures S2 and S3). Additionally, the strong vibronic transitions at 7.61, 7.70, and 8.14 eV can unambiguously be separated and identified by comparison with Franck–Condon (FC) simulations and calculated adiabatic ionization energies (AIE) using high-level quantum chemical methods (Figure 2, Figure S3, and Table 1). The first peak at 7.61 ± 0.03 eV corresponds to the undesired ionization of carbazole **2**, as confirmed by the clean ms-TPE spectrum (*m*/*z* 167) of non-pyrolyzed byproduct **2** (Figure S2), which is also in a good agreement with the previously reported value of 7.6 eV for carbazole **2**.^57^ Nevertheless, the comparison of the ms-TPE spectrum at *m*/*z* 166 with that obtained at *m*/*z* 167 and the FC factors of carbazole **2** suggests that no strong transitions are present above 7.65 eV (see Figure S3); hence, the observed peaks in the ms-TPE spectrum (*m*/*z* 166) are assigned solely to the ionization of *N*-carbazolyl radical **1**. The peak at 7.70 ± 0.03 eV is assigned to the adiabatic ionization energy (AIE) of radical **1** in its π (^2^B_1_) ground state to nitrenium cation **1**^**+**^ in its closed-shell singlet (^1^A_1_) ground state. The subsequent peak at 8.14 ± 0.03 eV corresponds to the first excited state of cation **1**^+^, the triplet (^3^B_2_) state. The spectral pattern is reasonably reproduced by FC simulations of the respective vibronic transitions at 800 K (Figure 2). In good agreement, AIEs to the ^1^A_1_ and ^3^B_2_ states of nitrenium ion **1**^**+**^ at around 7.8 and 8.1 eV are systematically predicted with composite methods and DFT calculations (Table 1). An experimental singlet–triplet energy gap of −0.44 ± 0.03 eV (10.1 ± 0.6 kcal/mol) is derived for cation **1**^**+**^, in excellent agreement with the calculations. Additional small peaks between 8.2 and 8.4 eV could be attributed to vibronic transitions of the triplet (^3^B_2_) state of cation **1**^**+**^ but also tentatively to the close-lying open-shell singlet excited state. Multiconfigurational methods such as CASSCF and NEVPT2 predict the open-shell singlet (^1^B_2_) state to be slightly higher in energy compared to the triplet (^3^B_2_) state of cation **1**^**+**^ (Figure S4 and Table S1). However, it was not possible to obtain reliable geometries and Hessian matrixes to simulate the vibrational structure of the open-shell singlet state. The short lifetime of these excited states usually results in broad, unstructured bands, which are difficult to identify.^58^ Nevertheless, we tentatively assign the band at 8.27 eV to the open-shell singlet state (^1^B_2_) state.

**Table 1:** Experimental and Calculated AIE of **1** in eV

method	**1**^+^(^1^A_1_) ← **1**	**1**^+^(^3^B_2_) ← **1**	Δ*E*_S–T_ (**1**^+^)
ms-TPE (exp)	7.70 ± 0.03	8.14 ± 0.03	–0.44
ωB97XD/6-311++G**	7.90	8.19	–0.29
B3LYP/6-311++G**	7.51	7.84	–0.33
CAM-B3LYP/6-311++G**	7.82	8.10	–0.28
M06-2X/6-311++G**	7.83	8.23	–0.40
CCSD(T)/ATZ//DZ^a^	7.58	8.02	–0.44
CBS-QB3	7.77	8.16	–0.39
G4	7.78	8.18	–0.40

a Geometry optimized at CCSD(T)/cc-pVDZ, while energy refined with aug-cc-pVTZ.

### Electronic Structure of Radical **1** and Cation **1**^+^

The *N*-carbazolyl radical **1** was optimized in various electronic states within *C*_2*v*_ symmetry. The ground state of radical **1** is predicted to be ^2^B_1_, with the unpaired electron residing in the out-of-plane b_1_ orbital at the bridged *N*-center, similar to the diphenylaminyl^59^ and fluorenyl radical.^60^ Hence, radical **1** is a highly delocalized π-radical. In contrast, the ^2^A_1_ state or σ-radical, with an unpaired electron localized in the in-plane a_1_ orbital at the *N*-center, is predicted to lie 35.9 kcal/mol higher in energy at the M06-2X/6-311++G** level of theory and should not be accessible in any phase.

**Figure 3:**
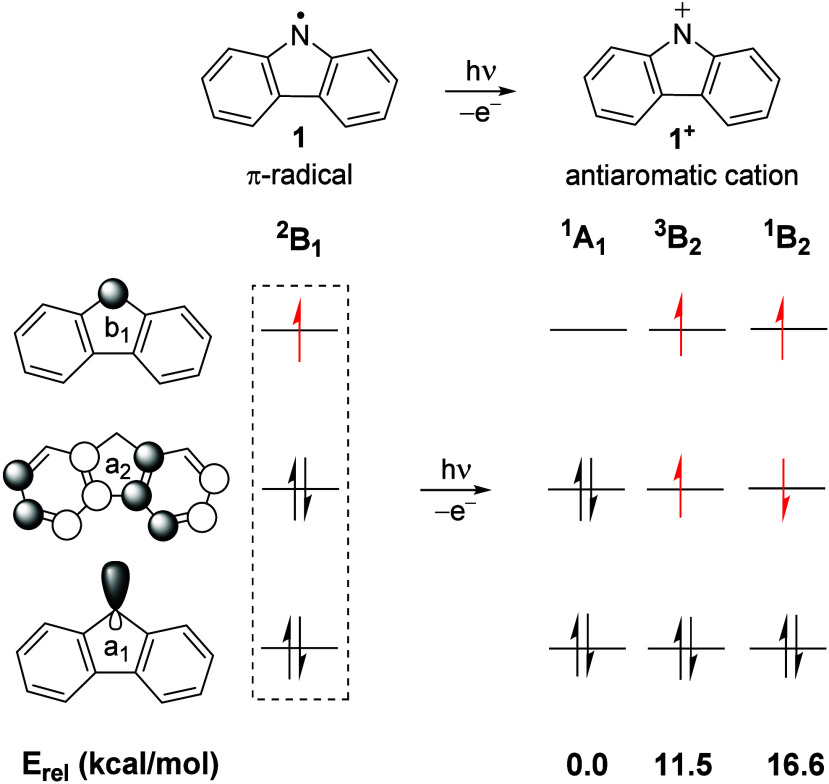
Relative energies of nitrenium ion **1**^**+**^ in different electronic states at the NEVPT2(15/14,14)/aug-cc-pVTZ//CASSCF(15/14,14)/aug-cc-pVTZ level of theory.

Ionization from the singly occupied molecular orbital (SOMO) of radical **1** yields closed-shell singlet state cation **1**^**+**^, while the open-shell singlet and triplet states of **1**^**+**^ are formed by ionization from the doubly occupied orbital (HOMO–1). The ground state of cation **1**^**+**^ is the closed-shell singlet (^1^A_1_), followed by the triplet (^3^B_2_) and the open-shell singlet (^1^B_2_) states which lie 11.5 and 16.6 kcal/mol higher in energy, respectively (Figure 3). The closed-shell singlet (^1^A_1_) state of **1**^**+**^ is formally an antiaromatic π-cation with a doubly occupied a_1_ (σ) and an empty b_1_ (π) orbitals at the bridged *N*-center.^39^ In contrast, the open-shell singlet (^1^B_2_) and triplet (^3^B_2_) cation with a (b_1_)^1^(a_2_)^1^ configuration resembles a π,π-diradical, with unpaired electrons at the *N*-center and the central five-member ring.

**Figure 4:**
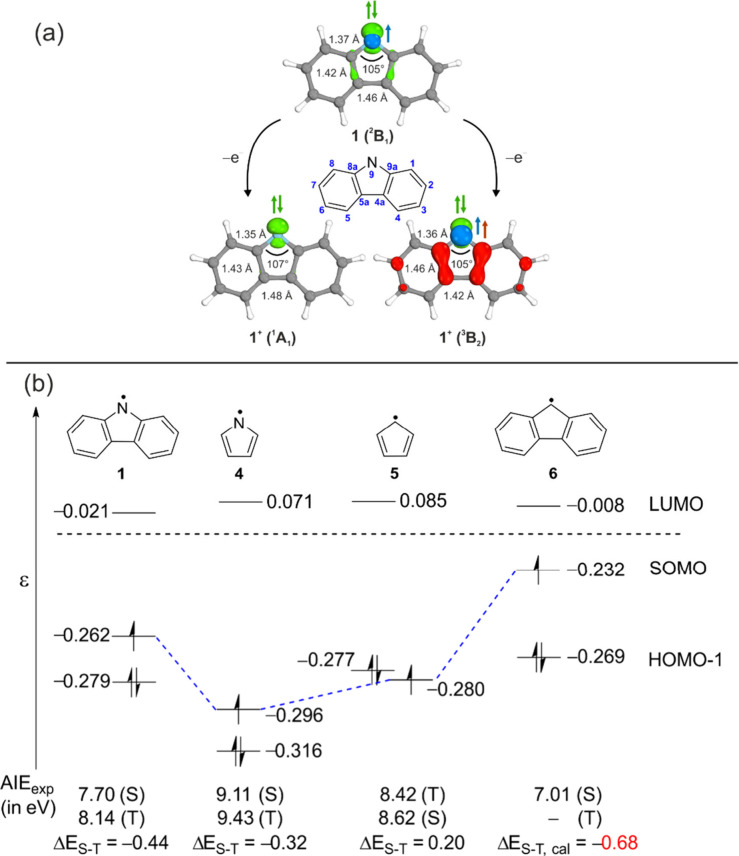
(a) Geometry and occupied orbitals (σ and π) of the lowest-energy states of radical **1** and singlet and triplet cation **1**^**+**^, optimized at the M06-2X/6-311++G** level of theory. Selected C–C bond distances and angles are shown. (b) Orbital energy diagram of *N*-carbazolyl **1**, pyrrolyl **4**, cyclopentadienyl **5**, and fluorenyl radical **6**. Absolute energies of the MOs are given in hartrees and calculated at the M06-2X/6-311++G**level of theory. Calculated Δ*E*_S–T_ (red color) with the G4 method.

Due to the rigid framework, the geometries of the singlet and triplet cations **1**^**+**^ and radical **1** are similar, which results in strong FC factors and intense vibronic transitions. The C_8a_–N_9_–C_9a_ angle in radical **1** is similar to that of singlet fluorenylidene due to the strong electron–electron repulsion of the nitrogen lone pair with the adjacent C–N bonds. Nevertheless, small changes in bond lengths are predicted in the heterocyclic core of **1** upon ionization to **1**^**+**^. The singlet state contains a pyrrolyl core, with shorter C_8a_–C_5a_ and C_9a_–C_4a_ bonds and a longer C_4a_–C_5a_ bond, whereas the reverse trend is predicted for the triplet state, resembling a substituted 3-pyrroline (Figure 4a).

The molecular orbital ordering and experimental AIEs of radical **1** and structurally related *C*_2*v*_-symmetric radicals (pyrrolyl **4**, cyclopentadienyl **5**, and fluorenyl **6**) were systematically compared and analyzed (Figure 4b). The AIE of **1** (7.70 eV) is experimentally found to be higher than that of its carbon analogue, fluorenyl radical **6** (7.01 eV),^60^ but significantly lower than that of the *N*-centered pyrrolyl radical **4** (9.11 eV).^61^ One-electron removal (ionization) of these radicals formally leads to the corresponding cations, **1**^**+**^, **4**^**+**^, **5**^**+**^, and **6**^**+**^. The ground state multiplicity of the cations depends on the orbital ordering in the corresponding radicals. The singly occupied molecular orbital (SOMO) is higher in energy than the doubly occupied orbitals in radicals **1**, **4**, and **6**; hence, cations **1**^**+**^, **4**^**+**^, and **6**^**+**^ exhibit a closed-shell singlet ground state. In contrast, SOMO–HOMO inversion (a formal violation of the Aufbau principle)^62^ is present in radical **5**, leading to cation **5**^**+**^ with a diradical character and triplet ground state.^63^ According to Baird’s rule, the triplet state of cation **5**^**+**^ is then classified as aromatic, in contrast to the strongly antiaromatic and first excited state, the singlet cation.

## Conclusion

In conclusion, pyrolysis of *N*-nitrosocarbazole (**3**) yields the highly delocalized *N*- carbazolyl π-radical **1**. Ionization of radical **1** via tunable VUV synchrotron radiation, combined with the double-imaging photoelectron photoion coincidence (i^2^PEPICO) technique, has enabled the detection and recording of the photoelectron spectra of radical **1** for the first time. Adiabatic ionization energies (AIEs) of radical **1** are measured at 7.70 ± 0.03 and 8.14 ± 0.03 eV, corresponding to the vibronic transitions from radical **1** to cation **1**^+^ in its singlet (^1^A_1_) and triplet (^3^B_2_) state. Cation **1**^+^ is a highly reactive antiaromatic species with a closed shell singlet ground state, while the first excited triplet (^3^B_2_) state lies 0.44 eV higher in energy. Comparison of the experimental AIEs of structurally related radicals **1**, **4**, **5**, and **6** reveals that (i) higher AIEs are found for *N*-centered radicals with respect to *C*-centered radicals and (ii) additional ring delocalization/conjugation lowers the AIE of both *N*- and *C*-centered radicals.^64^ These AIE shifts result from the stabilization of the singly occupied molecular orbital (SOMO) localized on the more electronegative nitrogen atom but also due to the destabilization of the SOMO upon increasing the π-conjugation (**5** → **6**, Figure 4b).

The ionization energy is a fundamental property of molecules to isomer-selectively identify reactive species in complex reaction mixtures and is used to derive enthalpies of formation for radicals and ions in charge transfer reactions. Along with the experimental and simulated ms-TPE spectra of **1**, the AIEs are tabulated in the PhotoElectron PhotoIon Spectral COmpendium (PEPISCO) database.^41^ In the regions of space dominated by photodissociation, the charge transfer reaction plays an important role in nitrogen chemistry. This valuable information is also relevant in astrochemical modeling and reliable reference data for the study of nitrogen-containing polycyclic aromatic hydrocarbons.

## Supporting Information Available

VMI results, ms-TPE spectra of carbazole **2** (*m*/*z* 167), combined ms-TPE spectra of *N*-carbazolyl radical **1** (*m*/*z* 166) and carbazole **2** (*m*/*z* 167), calculated excited states, AIEs of radical **1**, molecular orbitals of **1**, and optimized geometries (PDF)
