# Supplementary material for: N-Carbazolyl π-Radical and Its Antiaromatic Nitrenium Ion: A Threshold Photoelectron Spectroscopic Study
Source: J Phys Chem A. 2024 Oct 26;128(45):9747–53. doi: 10.1021/acs.jpca.4c05855 (PMC11571212; doi:10.1021/acs.jpca.4c05855)
Supplement: Supplementary file 1 — jp4c05855_si_001.pdf [file jp4c05855_si_001.pdf]

N-Carbazolyl π-Radical and Its Antiaromatic Nitrenium Ion:

A Threshold Photoelectron Spectroscopic Study

Mayank Saraswat[1], Adrian Portela-Gonzalez[1], Enrique Mendez-Vega[1], Wolfram Sander[1*] and Patrick Hemberger[2*]

[1]Lehrstuhl für Organische Chemie II, Ruhr-Universität Bochum, Bochum 44780, Germany.
E-mail: wolfram.sander@rub.de

[2]Laboratory for Synchrotron Radiation and Femtochemistry, Paul Scherrer Institut (PSI), Villigen CH-5232, Switzerland. E-mail: patrick.hemberger@psi.ch

Table of Contents

Velocity Map Imaging 2
ms-TPE Spectra of the FVP Products 3
Excited State Calculations..5
Cartesian Coordinates 7

Velocity Map Imaging

The velocity map image at 550 K (Figure S1) shows a narrow molecular beam component. In addition, the mass spectrum with pyrolysis off at 8.5 eV (Figure 1) does not show any signal at m/z 166. Moreover, full conversion is achieved at 800 K, which renders a contamination of dissociative ionization of the precursor of the ms-TPES at m/z 166 negligible.

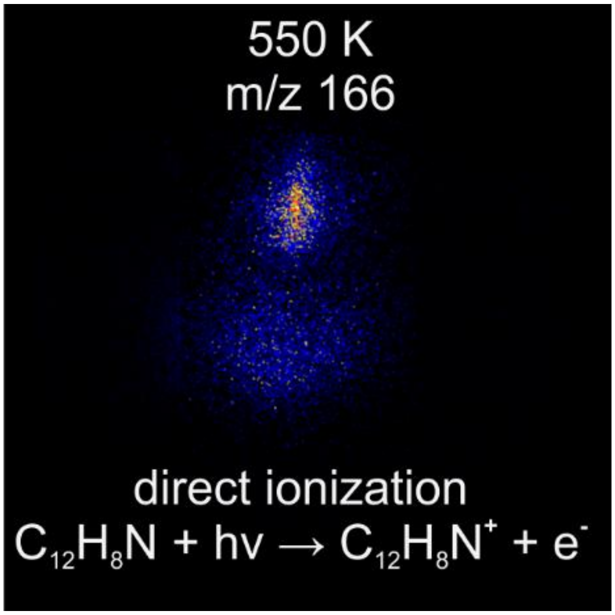

Figure S1. Ion velocity map imaging allows for distinguishing the molecular beam from the room temperature background formed via collisions within the vacuum chamber, as well as differentiating between direct ionization and dissociative ionization based on the kinetic energy release of the fragments. This technique is essential when investigating reactive and open-shell molecules. The selected ion images show the direct ionization of 9-carbazolyl radical 1 (m/z 166) at 8.5 eV.

ms-TPE Spectra of the FVP Products

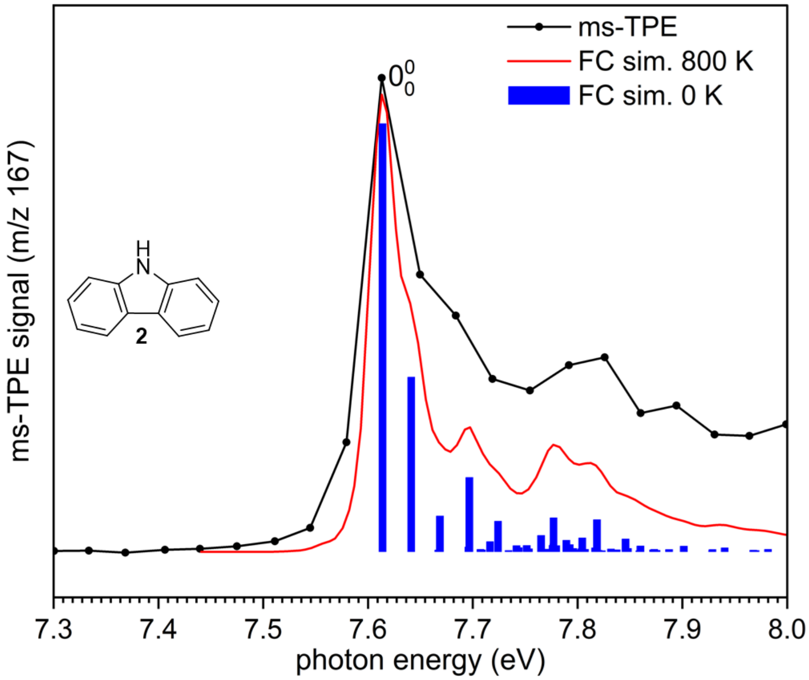

Figure S2. Comparison of the ms-TPE spectrum of m/z 167 recorded after FVP of *N*-nitrosocarbazole **3** at 800 K (black trace) with Franck–Condon (FC) simulations of carbazole **2**. Spectral simulations were performed at 0 K (blue sticks) and at 800 K (red trace) by convolution with 25 meV fwhm Gaussians over the vibrational frequencies computed with M06-2X/6-311++G** level of theory.

The spectrum of m/z 166 shows a large signal at 7.61 eV that clearly corresponds to carbazole (m/z 167). The integrated area of m/z 166 was decreased to avoid contamination of the signal from m/z 167. This attempt was unsuccessful, resulting in a lower resolution for the rest of the spectrum of m/z 166 due to a decrease in signal intensity. The spectrum of m/z 166 was refined by subtracting the spectrum of m/z 167, assuming that the signal at 7.61 eV (IP of carbazole) observed in the spectrum of m/z 166 corresponds to carbazole. Additional subtractions with a lower influence from the spectrum of m/z 167 were attempted, although the best resolution was achieved by matching the intensity of the spectrum at m/z 167 to the photon counts at 7.61 eV in the spectrum of m/z 166. The spectra that were subtracted are shown in Figure S3, and the resulting subtracted spectrum was used as the experimental spectrum of m/z 166 for fitting (Figure 2).

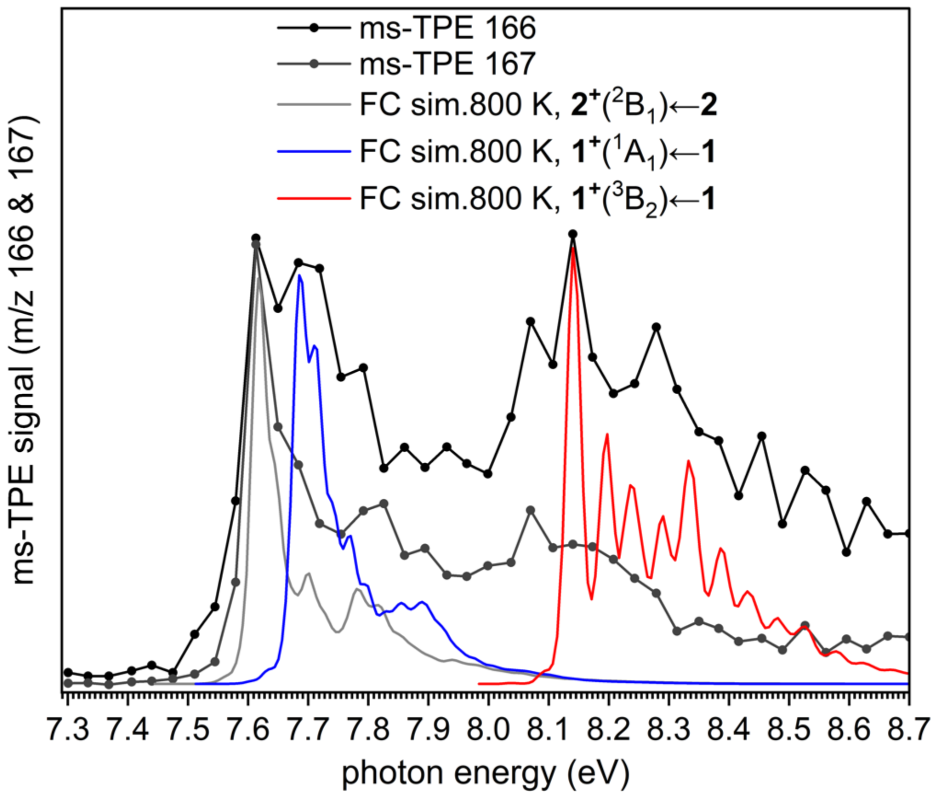

Figure S3. Comparison of the ms-TPE spectra of the signals at m/z 166 (contaminated with 167 in black trace) and 167 (grey trace), recorded upon FVP of precursor **3** at 800 K, with Franck−Condon (FC) simulations at 800 K of the vibronic transitions of 9-carbazolyl radical **1** to cation $\mathbf{1}^+$ in its $^1A_1$ (blue trace) and $^3B_2$ (red trace) electronic states, as well as the ionization of carbazole **2** (grey trace) is shown. FC simulations are convoluted using 25 meV fwhm Gaussians over the vibrational frequencies computed with M06-2X/6-311++G** level of theory.

Excited State Calculations

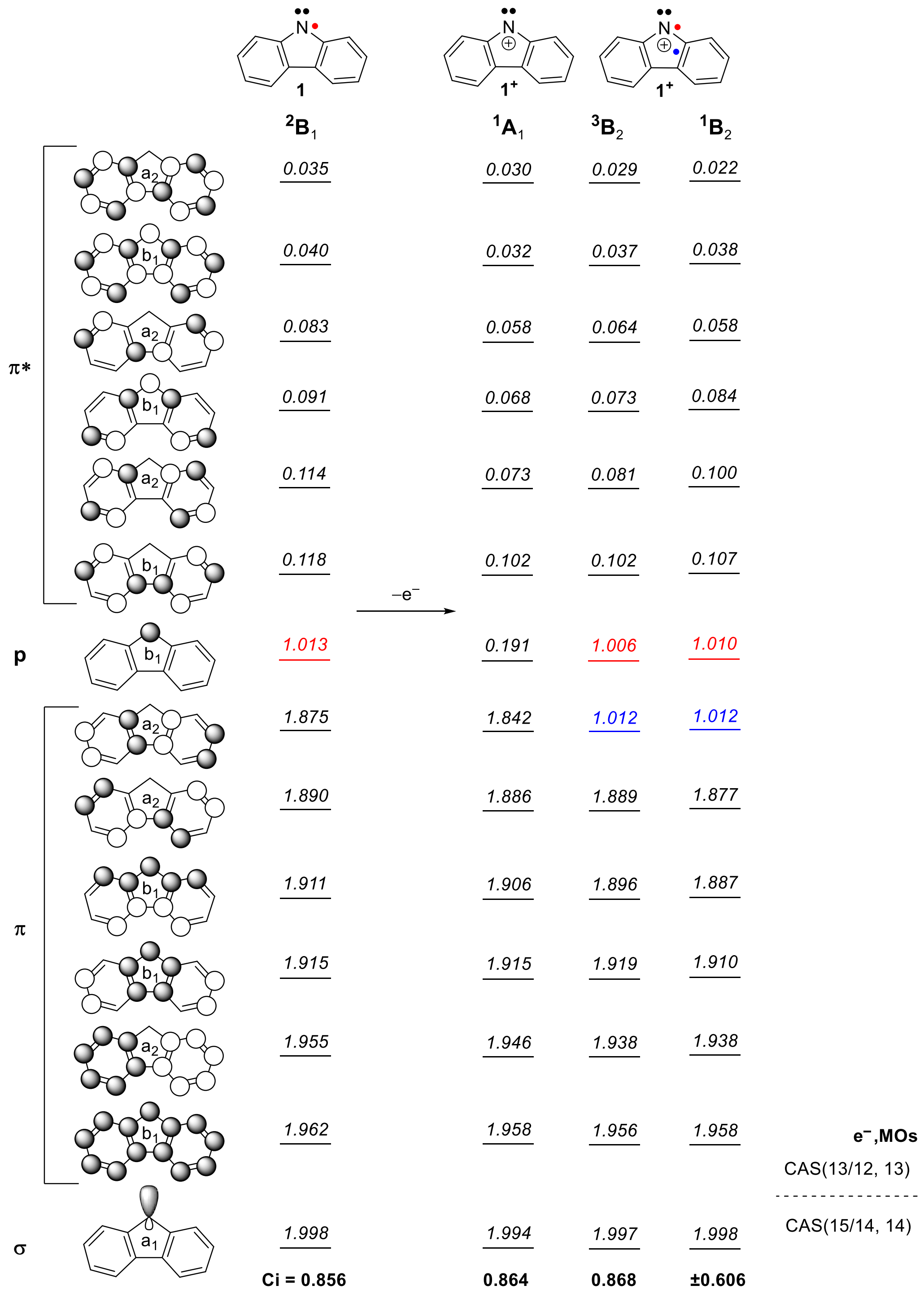

Figure S4. Orbital energy diagram for the leading configurations of the *N*-carbazolyl radical **1** and its cation $\mathbf{1}^+$ in the lowest-energy electronic states optimized at the CASSCF(15/14,14)/aug-cc-pVTZ level of theory. A reduced complete active space CAS(e^-,MOs) is also used excluding the low-lying σ orbital, denoted CAS(13,13) for **1** and CAS(12,13) for $\mathbf{1}^+$. Occupation numbers of natural orbitals and Ci coefficient of the leading configuration in the reference wavefunction are shown. The open-shell singlet $^1B_2$ state is described by two configurations with the same weight but alternating spins.

Table S1. Calculated Adiabatic Ionization Energies (AIE) from 1 to 1^+ in its Lowest-Energy Electronic States (in eV)

Method	$^1A_1 \leftarrow {}^2B_1$	$^3B_2$	$^1B_2$
M06-2X/6-311++G*	7.81	+0.45	–
M06-2X/6-311++G* + ZPE	7.83	+0.40	–
CASSCF(13/12,13)/aug-cc-pVTZ	6.79	+0.38	+0.47
CASSCF(15/14,14)/aug-cc-pVTZ	6.75	+0.39	+0.49
NEVPT2(13/12,13)/aug-cc-pVTZ // CASSCF(13/12,13)/aug-cc-pVTZ	7.41	+0.47	+0.70
NEVPT2(15/14,14)/aug-cc-pVTZ // CASSCF(15/14,14)/aug-cc-pVTZ	7.39	+0.50	+0.72
CCSD(T)/cc-pVDZ	7.27	+0.46	–
CCSD(T)/aug-cc-pVTZ // CCSD(T)/cc-pVDZ	7.54	+0.44	–
CBS-QB3	7.77	+0.39	–
G4	7.78	+0.40	–
Stationary Point	Min.	Min.	–

Calculated AIE from *N*-carbazolyl radical **1** to cation $\mathbf{1}^+$ in its closed-shell singlet ($^1A_1$) ground state. Calculated relative energies of the lowest-energy excited states of $\mathbf{1}^+$ with respect to the $^1A_1$ state. The open-shell singlet ($^1B_2$) state is only calculated with multiconfigurational methods. Composite calculations include zero-point energy (ZPE) corrections.

Cartesian Coordinates

Optimized geometries at the M06-2X/6-311++G** level of theory.

2 ($^1A_1$)				2^+ ($^2B_1$)			
C	0.00000000	-3.03147100	-1.15042105	C	0.00000000	3.05302800	-1.11793908
C	0.00000000	-3.41523600	0.19751495	C	0.00000000	3.42857800	0.22675792
C	0.00000000	-2.47285300	1.21642795	C	0.00000000	2.46938000	1.23078192
C	0.00000000	-1.12958200	0.85383595	C	0.00000000	1.13056700	0.82937892
C	0.00000000	-0.72255800	-0.50212305	C	0.00000000	0.73066600	-0.53348408
C	0.00000000	-1.69072700	-1.50570205	C	0.00000000	1.69853200	-1.51088608
H	0.00000000	-3.79243500	-1.91994605	H	0.00000000	3.81997600	-1.88091208
H	0.00000000	-4.46793200	0.45038395	H	0.00000000	4.47727600	0.48906992
H	0.00000000	-2.77454800	2.25647695	H	0.00000000	2.74630100	2.27685592
H	0.00000000	-1.39827000	-2.54840505	H	0.00000000	1.43953200	-2.56117208
C	-0.00000000	1.12958200	0.85383595	C	0.00000000	-1.13056700	0.82937892
C	-0.00000000	2.47285300	1.21642795	C	0.00000000	-2.46938000	1.23078192
C	-0.00000000	3.41523600	0.19751495	C	0.00000000	-3.42857800	0.22675792
C	-0.00000000	3.03147100	-1.15042105	C	0.00000000	-3.05302800	-1.11793908
C	-0.00000000	1.69072700	-1.50570205	C	0.00000000	-1.69853200	-1.51088608
C	-0.00000000	0.72255800	-0.50212305	C	0.00000000	-0.73066600	-0.53348408
H	-0.00000000	2.77454800	2.25647695	H	0.00000000	-2.74630100	2.27685592
H	-0.00000000	4.46793200	0.45038395	H	0.00000000	-4.47727600	0.48906992
H	-0.00000000	3.79243500	-1.91994605	H	0.00000000	-3.81997600	-1.88091208
H	-0.00000000	1.39827000	-2.54840505	H	0.00000000	-1.43953200	-2.56117208
N	0.00000000	0.00000000	1.65062295	N	0.00000000	0.00000000	1.60564492
H	0.00000000	0.00000000	2.65422695	H	0.00000000	0.00000000	2.61748792
**1 ($^2B_1$)**				**1^+ ($^1A_1$)**			
C	0.00000000	3.04738700	-1.09257491	C	0.00000000	3.07085100	-1.04926413
C	0.00000000	3.40420100	0.25548909	C	0.00000000	3.41523100	0.29395187
C	0.00000000	2.43174100	1.25125409	C	0.00000000	2.41772400	1.26927687
C	0.00000000	1.09179500	0.86181109	C	0.00000000	1.08754600	0.83098587
C	0.00000000	0.73234200	-0.51374891	C	0.00000000	0.74103100	-0.55607613
C	0.00000000	1.70247000	-1.49096091	C	0.00000000	1.71743400	-1.50092813
H	0.00000000	3.82484300	-1.84824091	H	0.00000000	3.86045100	-1.79264713
H	0.00000000	4.45304600	0.52861109	H	0.00000000	4.45833500	0.58226687
H	0.00000000	2.69224700	2.30300609	H	0.00000000	2.64399100	2.32917887
H	0.00000000	1.44461700	-2.54436891	H	0.00000000	1.50435300	-2.56287513
C	0.00000000	-1.09179500	0.86181109	C	0.00000000	-1.08754600	0.83098587
C	0.00000000	-2.43174100	1.25125409	C	0.00000000	-2.41772400	1.26927687
C	0.00000000	-3.40420100	0.25548909	C	0.00000000	-3.41523100	0.29395187
C	0.00000000	-3.04738700	-1.09257491	C	0.00000000	-3.07085100	-1.04926413
C	0.00000000	-1.70247000	-1.49096091	C	0.00000000	-1.71743400	-1.50092813
C	0.00000000	-0.73234200	-0.51374891	C	0.00000000	-0.74103100	-0.55607613
H	0.00000000	-2.69224700	2.30300609	H	0.00000000	-2.64399100	2.32917887
H	0.00000000	-4.45304600	0.52861109	H	0.00000000	-4.45833500	0.58226687
H	0.00000000	-3.82484300	-1.84824091	H	0.00000000	-3.86045100	-1.79264713
H	0.00000000	-1.44461700	-2.54436891	H	0.00000000	-1.50435300	-2.56287513
N	0.00000000	0.00000000	1.69525009	N	0.00000000	0.00000000	1.63325687

**T-1⁺ (³B₂)**			
C	0.00000000	3.00952400	-1.11171900
C	0.00000000	3.36893200	0.26852000
C	0.00000000	2.42311500	1.27290600
C	0.00000000	1.08016700	0.88310100
C	0.00000000	0.71135900	-0.52630700
C	0.00000000	1.68900800	-1.51980500
H	0.00000000	3.80088100	-1.85175500
H	0.00000000	4.42196200	0.52524200
H	0.00000000	2.69151600	2.32154800
H	0.00000000	1.43079200	-2.57158000
C	0.00000000	-1.08016700	0.88310100
C	0.00000000	-2.42311500	1.27290600
C	0.00000000	-3.36893200	0.26852000
C	0.00000000	-3.00952400	-1.11171900
C	0.00000000	-1.68900800	-1.51980500
C	0.00000000	-0.71135900	-0.52630700
H	0.00000000	-2.69151600	2.32154800
H	0.00000000	-4.42196200	0.52524200
H	0.00000000	-3.80088100	-1.85175500
H	0.00000000	-1.43079200	-2.57158000
N	0.00000000	0.00000000	1.70753400

Optimized geometries at the CASSCF(15/14,14)/aug-cc-pVTZ level of theory.

**1** ($^2B_1$)				$\mathbf{1}^+$ ($^1A_1$)			
C	0.00000000	-1.70249669	-1.3536941526	C	0.00000000	-1.71684997	-1.3726993664
C	0.00000000	-0.73309173	-0.3769967279	C	0.00000000	-0.73532158	-0.4325872001
C	0.00000000	-1.09268273	0.9967975556	C	0.00000000	-1.08145439	0.9554123501
C	0.00000000	-2.42928378	1.3901541075	C	0.00000000	-2.40709325	1.3955202321
C	0.00000000	-3.40573298	0.3925759090	C	0.00000000	-3.41247481	0.4215266559
C	0.00000000	-3.05205686	-0.9555747926	C	0.00000000	-3.07643100	-0.9214209609
H	0.00000000	-1.44955965	-2.3966137285	H	0.00000000	-1.50956487	-2.4241652604
H	0.00000000	-2.69027620	2.4298984862	H	0.00000000	-2.62950661	2.4439212366
H	0.00000000	-4.44332016	0.6650024032	H	0.00000000	-4.44247941	0.7143345670
H	0.00000000	-3.82147820	-1.7031527144	H	0.00000000	-3.85755519	-1.6558551076
C	0.00000000	1.70249669	-1.3536941526	C	0.00000000	1.71684997	-1.3726993664
C	0.00000000	3.05205686	-0.9555747926	C	0.00000000	3.07643100	-0.9214209609
C	0.00000000	3.40573298	0.3925759090	C	0.00000000	3.41247481	0.4215266559
C	0.00000000	2.42928378	1.3901541075	C	0.00000000	2.40709325	1.3955202321
C	0.00000000	1.09268273	0.9967975556	C	0.00000000	1.08145439	0.9554123501
C	0.00000000	0.73309173	-0.3769967279	C	0.00000000	0.73532158	-0.4325872001
H	0.00000000	1.44955965	-2.3966137285	H	0.00000000	1.50956487	-2.4241652604
H	0.00000000	3.82147820	-1.7031527144	H	0.00000000	3.85755519	-1.6558551076
H	0.00000000	4.44332016	0.6650024032	H	0.00000000	4.44247941	0.7143345670
H	0.00000000	2.69027620	2.4298984862	H	0.00000000	2.62950661	2.4439212366
N	0.00000000	0.00000000	1.8232043093	N	0.00000000	0.00000000	1.7520227077

**1**$^+$ ($^3B_2$)				**1**$^+$ ($^1B_2$)			
C	0.00000000	-1.69066585	-1.3812446054	C	0.00000000	-1.66688891	-1.3658876099
C	0.00000000	-0.71103664	-0.3887795244	C	0.00000000	-0.69948096	-0.3515151115
C	0.00000000	-1.07735572	1.0127235533	C	0.00000000	-1.08629173	1.0463150558
C	0.00000000	-2.42317138	1.4108285923	C	0.00000000	-2.42522797	1.4109479700
C	0.00000000	-3.37127203	0.4141383087	C	0.00000000	-3.37186880	0.3802875505
C	0.00000000	-3.01088780	-0.9724985434	C	0.00000000	-2.99584641	-0.9852732478
H	0.00000000	-1.43691320	-2.4220678772	H	0.00000000	-1.38939651	-2.4010242617
H	0.00000000	-2.68356399	2.4494129119	H	0.00000000	-2.71661860	2.4411820424
H	0.00000000	-4.41241925	0.6685538855	H	0.00000000	-4.41536098	0.6240658510
H	0.00000000	-3.79432942	-1.7036409424	H	0.00000000	-3.76223178	-1.7341436433
C	0.00000000	1.69066585	-1.3812446054	C	0.00000000	1.66688891	-1.3658876099
C	0.00000000	3.01088780	-0.9724985434	C	0.00000000	2.99584641	-0.9852732478
C	0.00000000	3.37127203	0.4141383087	C	0.00000000	3.37186880	0.3802875505
C	0.00000000	2.42317138	1.4108285923	C	0.00000000	2.42522797	1.4109479700
C	0.00000000	1.07735572	1.0127235533	C	0.00000000	1.08629173	1.0463150558
C	0.00000000	0.71103664	-0.3887795244	C	0.00000000	0.69948096	-0.3515151115
H	0.00000000	1.43691320	-2.4220678772	H	0.00000000	1.38939651	-2.4010242617
H	0.00000000	3.79432942	-1.7036409424	H	0.00000000	3.76223178	-1.7341436433
H	0.00000000	4.41241925	0.6685538855	H	0.00000000	4.41536098	0.6240658510
H	0.00000000	2.68356399	2.4494129119	H	0.00000000	2.71661860	2.4411820424
N	0.00000000	0.00000000	1.8251454826	N	0.00000000	0.00000000	1.8700878093
